# The Association of Serum IL-33 and sST2 with Breast Cancer

**DOI:** 10.1155/2015/516895

**Published:** 2015-09-20

**Authors:** Zhi-Ping Yang, Dan-Yan Ling, Yong-Hong Xie, Wan-Xin Wu, Jin-Rui Li, Jin Jiang, Jia-Lian Zheng, Yao-Hua Fan, Ye Zhang

**Affiliations:** Oncology Department, The First Affiliated Hospital of Jiaxing University, Jiaxing 314001, China

## Abstract

Breast cancer is one of the most common malignant diseases in women. The main cause of death from breast cancer is its metastases at distant sites in the body. Interleukin-33 (IL-33) is a cytokine of the IL-1 family and found overexpressed in various cancers. The aim of the present study was to explore the association of serum IL-33 and sST2 with breast cancer. Here, the serum levels of Interleukin-33 (IL-33) and sST2 were found significantly higher in breast cancer patients than in healthy volunteers. Serum levels of vascular endothelial growth factor (VEGF), metalloproteinase-11 (MMP-11), and platelet-derived growth factor-C (PDGF-C) were also greater in breast cancer patients compared to healthy volunteers. We found that serum levels of IL-33 or sST2 were positively correlated with the serum levels of VEGF, MMP-11, and PDGF-C. Moreover, breast cancer dataset downloaded from The Cancer Genome Atlas showed that patients with higher level of MMP-11 or PDGF-C expression had shorter survival time than those with lower level of these proteins. In conclusion, IL-33 and sST2 may serve as noninvasive diagnosis markers for breast cancer. IL-33 and sST2 were significantly associated with MMP-11 or PDGF-C which indicated poor prognosis of breast cancer patients.

## 1. Introduction

Breast cancer is the second most common malignant tumor, accounting for approximately 14% of all neoplastic diseases, and it is the most frequent cause of death in women 20 to 59 years of age [1]. Thus, early diagnosis and effective therapies for breast cancer are urgently needed. In addition, the main cause of deaths from breast cancer is not the primary tumor itself but is its metastases at distant sites in the body [2]. Improving our understanding of the metastatic process might improve clinical management of the disease.

Interleukin-33 (IL-33), a cytokine of the IL-1 family, was identified as a natural ligand for the ST2. Differential splicing of ST2 mRNA generated three different isoforms: a transmembrane form (ST2L), a soluble secreted form (sST2) [3], and a variant ST2 (ST2V) [4]. ST2L is a membrane-bound receptor for IL-33. The IL-33/ST2L axis stimulates the generation of cytokines and immunoglobulins characteristic of a type 2 immune response [5]. sST2, which lacks the transmembrane and intracellular domains, is regarded as a decoy receptor for IL-33 with anti-inflammatory properties [3], while ST2V mainly presents in the human gut [4]. It has been reported that IL-33 was overexpressed in various cancers. Elevated expression of IL-33 was reported in colorectal cancer (CRC) tissues [6] and serum of breast cancer [7] and non-small cell lung cancer (NSCLC) patients [8]. By using 4T1 breast cancer model, Jovanovic et al. demonstrated the role of time-dependent increase of endogenous IL-33 in primary tumors and metastatic lungs during cancer progression [9]. Moreover, high serum levels of sST2 were considered as a risk factor for breast cancer. Serum levels of sST2 were significantly higher in primary breast cancer patients than in healthy women [7] and notably higher in metastatic breast cancer patients than in primary breast cancer patients [10]. However, the detailed role of IL-33 and sST2 in the metastatic process of breast cancer has not been explored.

Invasion and angiogenesis are key steps of the metastatic process of breast cancer [2, 11]. Vascular endothelial growth factor (VEGF) [12, 13] and platelet-derived growth factor-C (PDGF-C) [14, 15] are important mediators of angiogenesis. Metalloproteinases (MMPs), including MMP-11, are able to degrade the extracellular matrix (ECM) and thus play an important role in tumor metastasis [16]. In the present study, we hypothesized that IL-33 was associated with invasion and angiogenesis of breast cancer and investigated whether serum IL-33 or sST2 was correlated with VEGF, PDGF-C, or MMP-11.

## 2. Materials and Methods

### 2.1. Serum Samples

From 2010 to 2012, 83 patients with breast cancer admitted to The First Affiliated Hospital of Jiaxing University were enrolled in this study. Sera samples were obtained from these patients before treatment. Sera from 83 age matched healthy volunteers with no evidence of illness were used as control samples. The control samples were obtained from screening clinics that were open to the general public during March 2012. All of the samples were obtained in the morning before food intake and were stored at −80°C until use.

This study was approved by the Ethics Committee of The First Affiliated Hospital of Jiaxing University. Informed and written consent was obtained from each individual according to the Ethics Committee guidelines.

### 2.2. Enzyme-Linked Immunosorbent Assay (ELISA) Analysis

Serum concentrations of IL-33, sST2, VEGF, MMP-11, and PDGF-C were measured with a commercially available sandwich enzyme-linked immunosorbent assay kit based on monoclonal antibodies (Bio-Swamp Life Science, Shanghai, China). Each sample was measured in duplicate. Assays were performed following the manufacturer's instructions. Plates were read at 450 nm using a microplate reader (Bio-Rad Laboratories Inc., Hercules, CA, USA). Accurate sample concentrations of the tested proteins were determined by comparing the specific absorbance with those obtained from the standards plotted on a standard curve.

### 2.3. Statistical Analysis

All statistical analyses were carried out using MedCalc software (Mariakerke, Belgium). The results were presented as the mean value ± SEM. Two-tailed Student's *t*-test was used to calculate the statistical significance of difference between groups. The relationships between two factors were assessed by Pearson correlation analysis. Breast invasive carcinoma (BRCA) dataset (version: 2014-08-22) was downloaded from The Cancer Genome Atlas (TCGA, https://tcga-data.nci.nih.gov/tcga/). Kaplan-Meier survival curve was conducted to evaluate the association between MMP-11 and PDGF-C mRNA level and survival rate of 930 patients with invasive breast cancer. Differences were considered significant with a value of *P* < 0.05.

## 3. Results

### 3.1. Serum Concentrations of IL-33, sST2, VEGF, MMP-11, and PDGF-C Assessed by ELISA

**Figure 1:**
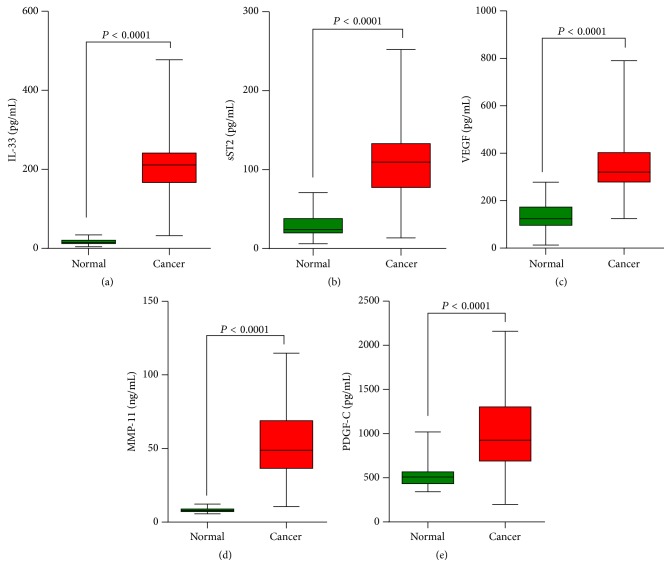
Analysis of the serum concentration of IL-33, sST2, VEGF, MMP-11, and PDGF-C in patients with breast cancer. The protein concentrations in the sera from healthy volunteers and patients were evaluated by ELISA. ELISA data are expressed as average protein concentration. The protein levels of these proteins were higher in the sera of patients with breast cancer than that in normal sera.

The protein concentrations of IL-33, sST2, VEGF, MMP-11, and PDGF-C were quantified in all specimens using ELISA (Figure 1). Sera concentration of IL-33 and sST2 was significantly higher in patients with breast cancer than in healthy volunteers (IL-33: 200.20 ± 9.35 pg/mL versus 16.34 ± 0.68 pg/mL, sST2: 104.30 ± 4.54 pg/mL versus 26.13 ± 1.20 pg/mL). These data indicated that the changes in the expression of IL-33/ST2 may be associated with breast cancer. Moreover, a significant difference of VEGF protein levels was also shown between healthy volunteers and breast cancer patients (349.40 ± 1.25 pg/mL versus 133.5 ± 5.70 pg/mL). MMP-11 and PDGF-C protein levels were remarkably higher in sera of breast cancer patients than in sera of healthy volunteers (MMP-11: 53.07 ± 2.63 ng/mL versus 8.16 ± 0.14 ng/mL, PDGF-C: 1023.00 ± 47.76 pg/mL versus 524.90 ± 15.01 pg/mL).

### 3.2. Correlation Analysis between the Serum Concentrations of IL-33 or sST2 and VEGF, MMP-11, or PDGF-C

**Figure 2:**
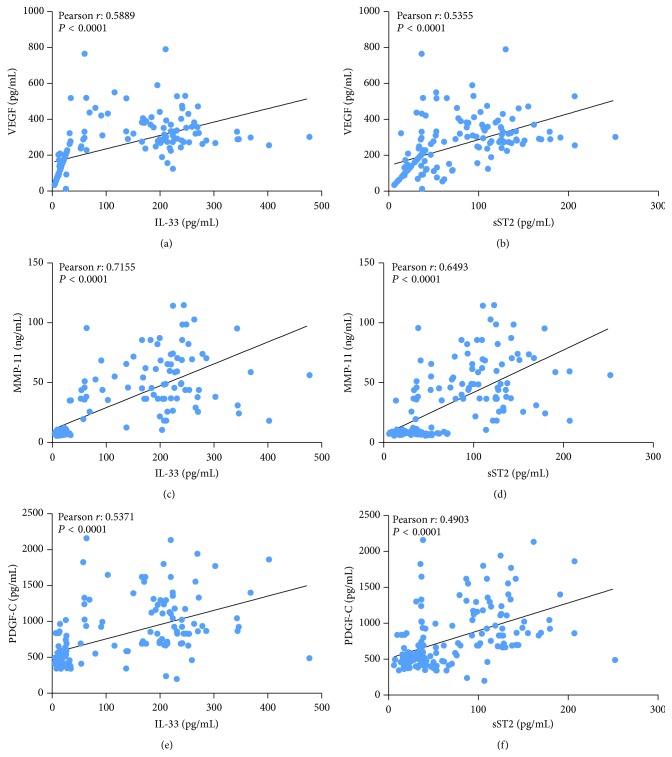
Correlation analysis of serum concentrations. The serum levels of two proteins were subjected to Pearson correlation analysis, which suggested a positive correlation between the two indicated proteins (*P* < 0.0001).

Pearson correlation analysis was carried out to determine the relationship between the serum concentrations of proteins (Figure 2). VEGF concentration strongly correlated with both IL-33 (*r* = 0.5889; *P* < 0.0001) and sST2 (*r* = 0.5355; *P* < 0.0001) concentration. MMP-11 and PDGF-C concentrations were also significantly associated with IL-33 (MMP-11: *r* = 0.7155, *P* < 0.0001; PDGF-C: *r* = 0.5171, *P* < 0.0001) and sST2 (MMP-11: *r* = 0.6493, *P* < 0.0001; PDGF-C: *r* = 0.4903, *P* < 0.0001) concentration.

### 3.3. MMP-11 and PDGF-C Expression Was Correlated with Poor Survival of Breast Cancer Patients

**Figure 3:**
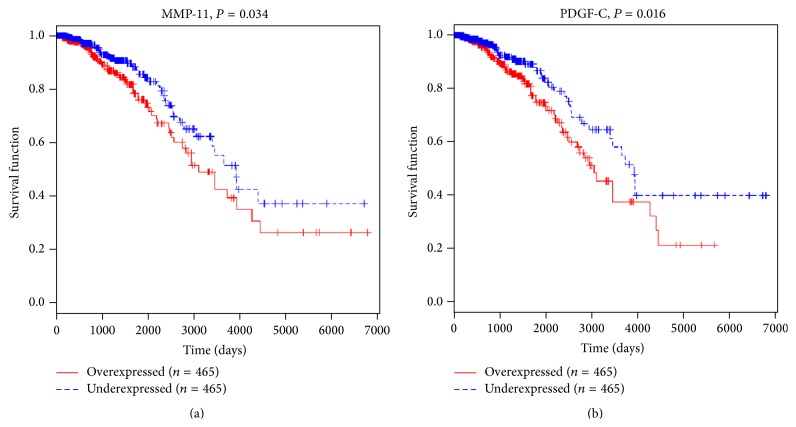
The survival time of high MMP-11 or PDGF-C expression level patients was notably shorter than that of low expression patients. Breast cancer dataset was downloaded from The Cancer Genome Atlas (TCGA).

To evaluate the clinical relevance of MMP-11 and PDGF-C mRNA in breast cancer in terms of prognosis, Kaplan-Meier survival analysis was performed on data downloaded from TCGA. Median values of MMP-11 and PDGF-C were assumed as cut-off for distinguishing low level from high level expression. Our results indicated that MMP-11 and PDGF-C mRNA was significantly associated with patient survival (Figure 3). Patients with low expression of these genes tended to have longer survival than patients with high levels of these genes (*P* < 0.05).

## 4. Discussion

Recently, the role of the IL-33/ST2 axis in the diagnosis, prognosis, or metastasis of cancers has been described [6–9]. Here, we found that the serum levels of IL-33 and sST2 were higher in patients with breast cancer than in healthy volunteers (Figure 1), suggesting that IL-33 and sST2 were associated with this disease.

Moreover, angiogenesis is now widely recognized as playing a pivotal role in the occurrence, development, and metastasis of tumors. VEGF, one of the most potent mediators of angiogenesis, stimulates the proliferation, migration, and invasion of endothelial cells [12, 13]. In solid tumors, the expression of VEGF indicates poor prognosis and a trend for metastasis [17, 18]. In the present study, the serum level of VEGF was notably increased in breast cancer patients compared to that in the healthy volunteers. The serum levels of IL-33 and sST2 were significantly correlated with the serum level of VEGF (Figure 2), which was consistent with a recent study reported by Lu et al. [7]. These data suggested an association of IL-33 and sST2 with angiogenesis.

Platelet-derived growth factor-C (PDGF-C) is a novel growth factor that binds to PDGF *α* and *β* receptor [14]. PDGF-C might serve as a transforming factor [15], a survival and mitogenic factor for tumor cells [19], or as a mitogenic and chemoattractant factor for cancer-associated fibroblasts [20, 21]. PDGF-C is also regarded as a critical regulator of pathological angiogenesis, which can promote tumor angiogenesis [16, 17]. Here, we analyzed the survival of breast cancer patients with higher or lower PDGF-C level based on data downloaded from TCGA. PDGF-C expression was a poor prognosis factor for breast cancer (Figure 3). Our ELISA results showed that PDGF-C level was elevated in the sera from patients with breast cancer (Figure 1) and that serum levels of IL-33 and sST2 in breast cancer patients had significant correlations with PDGF-C (Figure 2), which indicated the diagnosis and prognosis value of IL-33 and sST2.

Invasion is preceded by degradation of the extracellular matrix (ECM) to enable the penetration of tissue boundaries. ECM is mainly degraded through metalloproteinases (MMPs) and the urokinase plasminogen activator (uPA) system. MMPs mediate the proteolysis of ECM at the invadopodial front of invasive breast cancer cell lines [16]. It is showed that MMP-11 was expressed specifically by fibroblasts in breast carcinomas and not in their benign counterparts [22]. Ahmad et al. reported that focal expression of MMP-11 by breast carcinoma cells was attributed to epithelial-to-mesenchymal transition (EMT) [23]. Here, our ELISA data showed an elevation of MMP-11 in sera of breast cancer patients (Figure 1) and a significant association between MMP-11 and IL-33 or sST2 concentration was found (Figure 2). Moreover, MMP-11 [23] may be an independent prognostic factor for invasive breast cancer patients. In line with these findings, MMP-11 levels were significantly associated with breast cancer patients' survival based on Kaplan-Meier survival analysis performed on data downloaded from TCGA (Figure 3). These data suggested that IL-33 and sST2 were significantly associated with MMP-11 or PDGF-C that indicate poor prognosis.

Taken together, serum levels of IL-33, sST2, VEGF, MMP-11, and PDGF-C were higher in breast cancer patients than in healthy volunteers. IL-33 and sST2 were positively correlated with VEGF, MMP-11, and PDGF-C in breast cancer patients. Moreover, expression of MMP-11 or PDGF-C indicated poor prognosis of breast cancer. Thus, the elevation of serum concentration of IL-33 or sST2 may be a valuable indicator of poor prognosis in breast cancer. Understanding of the association of IL-33 and angiogenesis or invasion might help in the design of efficient and safe therapy for breast cancer. However, there are some limitations of our study including relative small sample size, no clinical characteristics analysis, and no follow-up of serum level before and after treatment. Further investigations based on more detailed clinical data are needed.
